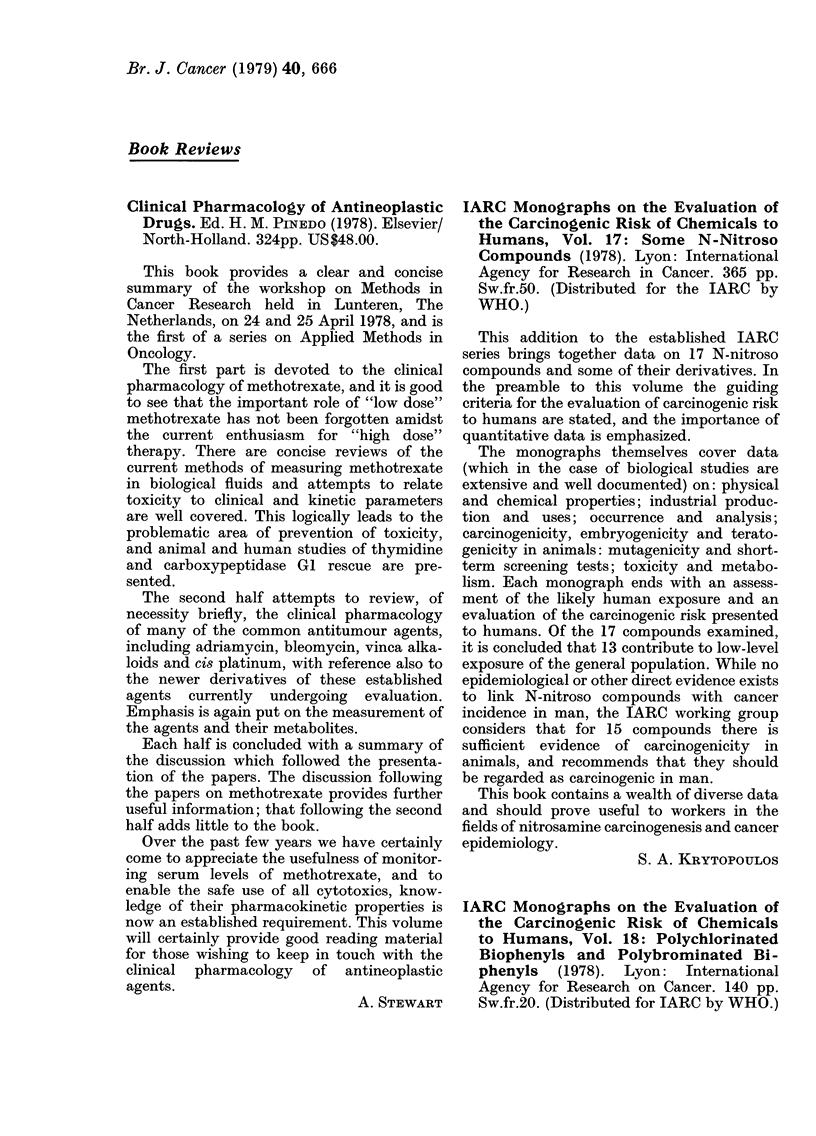# IARC Monographs on the Evaluation of the Carcinogenic Risk of Chemicals to Humans, Vol. 17: Some N-Nitroso Compounds (1978)

**Published:** 1979-10

**Authors:** S. A. Krytopoulos


**IARC Monographs on the Evaluation of the Carcinogenic Risk of Chemicals to Humans, Vol. 17: Some N-Nitroso Compounds** (1978). Lyon: International Agency for Research in Cancer. 365 pp. Sw.fr.50. (Distributed for the IARC by WHO.)

This addition to the established IARC series brings together data on 17 N-nitroso compounds and some of their derivatives. In the preamble to this volume the guiding criteria for the evaluation of carcinogenic risk to humans are stated, and the importance of quantitative data is emphasized.

The monographs themselves cover data (which in the case of biological studies are extensive and well documented) on: physical and chemical properties; industrial production and uses; occurrence and analysis; carcinogenicity, embryogenicity and teratogenicity in animals: mutagenicity and short-term screening tests; toxicity and metabolism. Each monograph ends with an assessment of the likely human exposure and an evaluation of the carcinogenic risk presented to humans. Of the 17 compounds examined, it is concluded that 13 contribute to low-level exposure of the general population. While no epidemiological or other direct evidence exists to link N-nitroso compounds with cancer incidence in man, the IARC working group considers that for 15 compounds there is sufficient evidence of carcinogenicity in animals, and recommends that they should be regarded as carcinogenic in man.

This book contains a wealth of diverse data and should prove useful to workers in the fields of nitrosamine carcinogenesis and cancer epidemiology.

S. A. KRYTOPOULOS